# Elective intraperitoneal procedures do not adversely impact paediatric ventriculoperitoneal shunt survival: 10-year retrospective cohort study

**DOI:** 10.1007/s00381-025-06963-6

**Published:** 2025-09-25

**Authors:** Tom Hoy, Annabelle M. Harbison, Robert A. J. Campbell, Liam G. Coulthard, Rachel E. Colbran, Michael J. Stuart

**Affiliations:** 1 Department of Neurosurgery, Townsville University Hospital, Townsville, QLD 4814 Australia; 2 Department of Neurosurgery, Queensland Children’s Hospital, South Brisbane, QLD 4101 Australia; 3 Faculty of Medicine, University of Queensland, Herston, Queensland 4006 Australia

**Keywords:** Hydrocephalus, Shunt, Laparotomy, Laparoscopy, Appendix, Paediatric

## Abstract

**Introduction:**

There is currently little evidence to guide the management of ventricular-peritoneal shunts during subsequent intraperitoneal procedures, and the influence of these procedures on the risk of shunt malfunction is poorly understood.

**Methods:**

A ten-year single institution retrospective analysis was undertaken to identify all paediatric patients with ventriculoperitoneal shunts. Statewide electronic medical records were reviewed to determine whether the patient had undergone any subsequent intraperitoneal procedures, and if so, how the shunt was managed during the procedure. Intraperitoneal procedures were divided into elective and emergency categories. Shunt survival was compared with patients not undergoing subsequent intraperitoneal procedures in time-dependent univariate and multivariate Cox proportional hazard models.

**Results:**

A total of 1084 shunt-related procedures were performed in 472 patients, of which 45 patients underwent elective and 15 patients underwent emergency intraperitoneal procedures during a mean follow-up of 4.85 years. The most common elective procedures were considered ‘clean’ procedures—gastrostomies 17 (38%) and hernia repairs 13 (29%), in addition to 8 (18%) ‘clean-contaminated’ colostomy or colectomy procedures. No significant association of elective intraperitoneal procedures with earlier shunt failure was found on univariate or multivariate analysis (HR 1.18, 95% CI 0.57–2.44, *p* = 0.66). Of patients presenting with an acute abdomen requiring surgical intervention, 4/15 (27%) were secondary to shunt infection, which increases to 4/6 (66%) in those without a clear preoperative alternative diagnosis.

**Conclusion:**

The performance of an elective intra-peritoneal procedure on a patient with a ventriculoperitoneal shunt in situ does not appear to increase the risk of subsequent shunt malfunction.

## Introduction

Hydrocephalus is a common issue in the paediatric neurosurgical population due to a number of causes including tumours, intraventricular haemorrhage of prematurity (IVHp), and congenital defects. Insertion of a ventriculoperitoneal shunt (VPS) remains the mainstay of treatment in most instances [1]. However, VPS malfunction and the need for VPS revision are significant causes of mortality and morbidity within this population, reinforcing the importance of preventative measures [2].

One area which remains poorly understood is the risk of performing an intraperitoneal procedure on a child who has a VPS in situ, and what strategies might mitigate that risk. This is a common situation given the association between certain neurosurgical conditions requiring a VPS and a comorbidities with the need for intraperitoneal procedures, for example, neurological injuries/lesions and dysphagia, IVHp, and other prematurity-associated issues such as inguinal hernias and congenital anomalies [3, 4]. Furthermore, young patients with shunts still have common unrelated abdominal procedures for conditions such as appendicitis [5].

The possible mechanisms of shunt failure extend beyond the obvious concern for acute infection from exposure to skin or colonic flora. These include direct injury to shunt tubing such as inadvertent incision, traction with disconnection or fracture, insufflation of gas with ‘air lock’, and delayed obstruction due to fibrosis around the catheter tip of inflammatory or low-virulence infective aetiology. Therefore, while previous efforts to address this question have primarily focussed on the outcome of short-term/acute infection, it is relevant to also consider the longer-term risk of diminished shunt survival [6, 7]. The scant previous analysis of longer-term shunt survival suffered from critical weaknesses such as very small sample size and inappropriate statistical analysis that do not consider the time-dependent nature of subsequent intraperitoneal procedures, which make the interpretation of those results challenging [8].

The primary aim of this study was to clarify with a larger sample and appropriate statistical methodology whether paediatric VPS patients who underwent elective intraperitoneal procedures were at risk of reduced shunt survival (time to shunt revision) during follow-up compared to those who did not. The secondary aim was to describe the management and outcomes of VPS at the time of emergency intraperitoneal procedures. 

## Methods

**Fig. 1:**
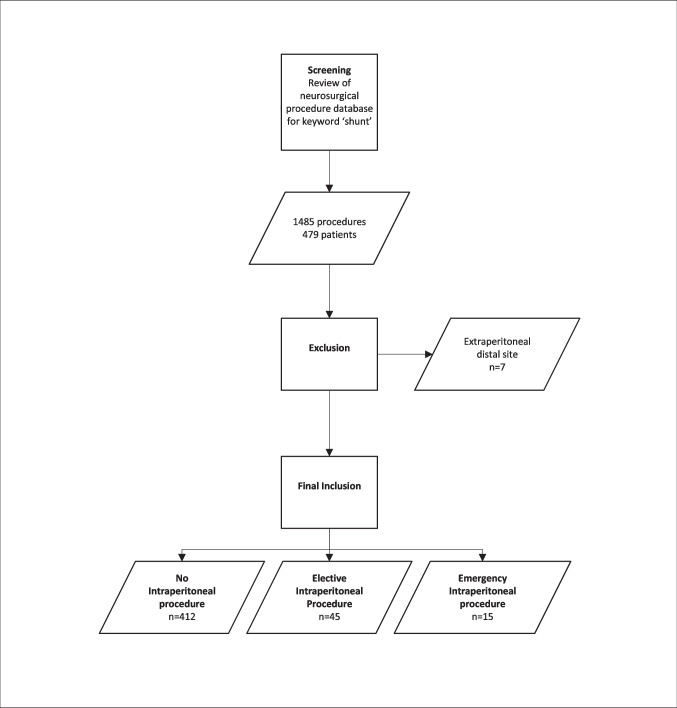
Case inclusion flowchart

A retrospective review of a prospectively maintained database was conducted, including all patients who underwent cerebrospinal fluid diversion procedures at the Queensland Children’s Hospital from 29 November 2014 to 1 September 2024. Patients undergoing insertion or revision of a ventricular shunt at that institution were captured in the database. For purposes of this study, patients were included if the first procedure was either a shunt insertion or revision. Due to the exclusivity of the paediatric hospital, patients were included from prematurity to 18 years of age. Patients with a non-intraperitoneal location of the distal shunt system (i.e. atrial or pleural) were excluded. Review of statewide electronic medical records was undertaken to detect any instances of intraperitoneal procedures undertaken via a laparoscopic or laparotomy approach. Intraperitoneal procedures included cases with minor peritoneal breach such as gastrostomy or hernia with opening of sac prior to reduction. Cases without documented peritoneal entry were not included (Fig. 1).

At the study institution, ventricular catheters are typically inserted with guidance from a neuronavigation system as standard of care, except in cases with very large ventricular calibre/thin cortical mantle. Peritoneal catheters are placed by mini-laparotomy/open cut-down as routine. CSF sampling is routinely undertaken from the ventricular catheter immediately after insertion and prior to connection to the valve. Intraventricular antibiotics are not routinely used. A standardized infection prevention protocol adapted from previously published recommendations was used for all cases [9].

Deaths due to intended palliation in the setting of progressive hydrocephalus, with confirmed hydrocephalus on neuroimaging or out of hospital cardiac arrest in a patient with a shunt, were defined as ‘shunt-related mortality’. Deaths due to palliation in the setting of a neoplasm were excluded from the definition of shunt-related mortality. Shunt related mortality was included as a ‘shunt revision’ in the survival analysis.

**Fig. 2:**
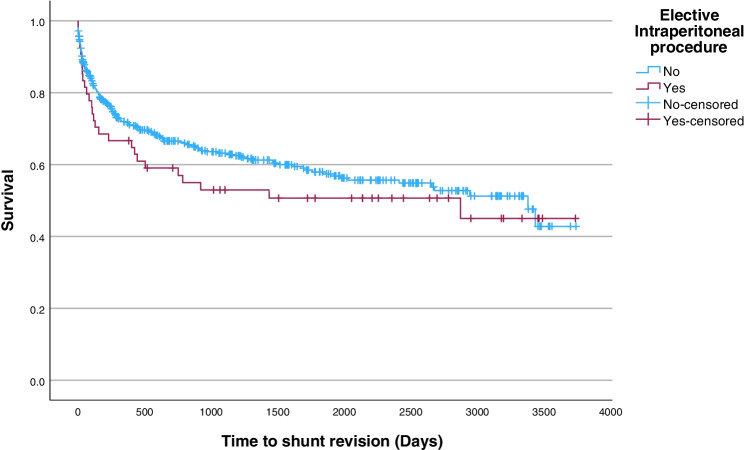
Survival curve for ventriculoperitoneal shunt patients with or without an elective intraperitoneal procedure

For survival analysis, cases were divided into intraperitoneal procedures scheduled in advance (elective) and cases performed due to an acute abdominal syndrome (emergency), irrespective of whether these were within the patient’s index admission (Fig. 2). This dichotomization was selected both to facilitate comparison with previous literature and due to the difference in potential contamination/inflammation in these contexts.

In instances where the low number of events precluded formal statistical analysis, descriptive statistics are presented. Where analysis is possible, univariate analysis of categorical variables was performed with the Pearson Chi-squared test. Student’s *T*-test was used to compare continuous data conforming to a normal distribution. Mann–Whitney *U* test was utilized for continuous data not conforming to a normal distribution. Due to the time-dependent nature of the subsequent intraperitoneal procedures, the influence of these on shunt survival was performed with a Cox proportional hazard model including the intraperitoneal procedure as a time-dependent covariate. Where patients have had more than one shunt procedure, the intervention prior to the intraperitoneal procedure was selected as the initial time point. Where patients had more than one intraperitoneal procedure, the first procedure was selected as the event time point. This was performed in a univariate manner and repeated in a multivariate model including factors found to be predictive of reduced shunt survival in previously published studies arising from this cohort and others [10–12]. Statistical significance was set at *p*< 0.05. All data analysis was performed using SPSS 30 (IBM Corporation, New York, 2024).

Ethical approval was obtained from the Queensland Children’s Hospital human research ethics committee (HREC/24/QCHQ/108644).

## Results

During the study period, 1084 shunt-related procedures were performed in 478 patients. Six of these patients had a shunt directed to an atrial or pleural distal site and were excluded from further follow-up (Fig. 1). The demographics reflect the developed-world setting of this study, with a mean age of 5.4 ± 5.7 years (range premature to 18) at the time of the index shunt procedure and a predominance of congenital (32%), neoplastic (29%), and haemorrhagic aetiologies. Post-infective hydrocephalus (5%) and idiopathic intracranial hypertension (2%) were rare pathologies in this cohort (Table 1).

**Table 1:** Demographics

Variable	No intraperitoneal procedure	Elective intraperitoneal procedure	Acute Intraperitoneal procedure	Significance
Age (Years) *				
	6.1 ± 5.6	4.4 ± 5.6	-	0.06
	6.1 ± 5.6	-	10.1 ± 6.4	0.01
Gender (female)	195 (47%)	16 (36%)	2 (13%)	0.01
Initial Shunt Procedure in database				
Shunt insertion	320 (78%)	34 (76%)	8 (53%)	0.29
Shunt revision	92 (22%)	11 (24%)	7 (47%)	
ASA score at initial procedure				
1	8 (2%)	0	0	0.79
2	111 (27%)	12 (27%)	6 (40%)	
3	270 (66%)	29 (64%)	8 (53%)	
4	23 (6%)	4 (9%)	1 (7%)	
Duration of initial procedure (minutes)*				
	98 ± 42	102 ± 43	-	0.55
	98 ± 42	-	95 ± 31	0.76
Indication for shunt insertion				0.23
Congenital (Including Chiari II)	126 (31%)	21 (47%)	4 (27%)	
Haemorrhage	108 (26%)	14 (31%)	5 (33%)	
Tumour	129 (31%)	7 (16%)	3 (20%)	
Infection	23 (6%)	1 (2%)	1 (7%)	
Idiopathic intracranial hypertension	9 (2%)	0	0	
Other	17 (4%)	2 (4%)	2 (13%)	
Shunt side				0.61
Right	350 (85%)	37 (82%)	13 (87%)	
Left	56 (14%)	8 (18%)	2 (14%)	
Bilateral	4 (1%)	0	0	
4th ventricle	2 (1%)	0	0	
Proximal site				0.02
Parietal-occipital	367 (89%)	37 (82%)	14 (93%)	
Frontal	39 (10%)	8 (18%)	1 (7%)	
Other	6 (2%)	0	0	
Valve				0.001
Medtronic Delta	252 (61%)	21 (47%)	5 (33%)	
Medtronic Strata	20 (5%)	5 (11%)	1 (7%)	
Orbus Sigma Valve	57 (14%)	31 (31%)	2 (13%)	
Codman Certas	58 (14%)	1 (2%)	2 (13%)	
Codman Hakim	1 (1%)	1 (2%)	1 (7%)	
Sophysa Polaris	14 (3%)	2 (4%)	2 (13%)	
Other Programmable	1 (1%)	0	0	
Other fixed	9 (2%)	1 (2%)	2 (13%)	
Total	412	45	15	472

^*^mean ± Standard deviation

*ASA* American Society of Anaesthesiologists

During the mean follow-up of 4.85 years (range 0–10), these patients underwent 45 elective and 15 emergency intraperitoneal procedures. The most common elective procedures were considered ‘clean’ procedures—gastrostomies 17 (38%), hernia repairs 13 (29%), and urological procedures 7 (16%). Clean-contaminated procedures such as the formation of an appendicocaecostomy for the administration of Malone Anterograde Continence Enemas (MACE) 4 (9%) and other colectomy/colostomy procedures 4 (9%) were less common. The emergency procedures performed comprised 7 (47%) appendicectomies, 2 (13%) cholecystectomies, and 6 (40%) exploratory procedures/adhesiolysis. Most elective procedures were performed years after shunt insertion (mean 2 years), and no subsequent intraperitoneal procedure was performed within 13 days of a shunt procedure. Only 4 elective procedures were performed within 30 days of a shunt procedure. During the elective procedures there were no specific precautions noted in the operation reports regarding the ventriculoperitoneal shunt in any case, while the shunt was externalized at the time of procedure in 4 (27%) of emergency procedures (Table 2). The survival analysis found no significant association of elective intraperitoneal procedures with earlier shunt failure on univariate analysis (HR 1.18, 95% CI 0.57–2.44, *p*= 0.66). This difference remained non-significant if only patients with ‘virgin’ unrevised shunts (*n*= 362) were included in the analysis (HR 1.33, 95% CI 0.57–3.10). There were some noted demographic differences between the cohorts in factors which have previously been associated with earlier shunt failure in this and other cohorts—younger age, a haemorrhagic aetiology of hydrocephalus, and the use of an Orbus Sigma Valve (OSV) [11]. In order to exclude collinearity with of these variables as an explanation for the lack of effect on shunt survival, the survival analysis was repeated in a multivariate Cox proportional hazard model with the inclusion of these additional variables. While there remained no association of elective intra-abdominal procedures with earlier shunt failure (HR 0.91, 95% CI 0.44–1.91, *p*= 0.80), older age remained a protective factor (HR 0.96, 95% CI 0.94–0.99, *p *= 0.015), and OSV valve a risk factor for earlier failure (HR 1.76, 95% CI 1.24–2.50, *p*= 0.002) (Table 3). Finally, this cohort includes a number of cases with only minor peritoneal entry such as gastrostomy (*n*= 17) and open hernia repairs (*n*= 13). When this analysis is repeated and these cases are excluded, there remains no significant association with earlier shunt failure.

**Table 2:** Intraperitoneal procedures and shunt management

Variable	Elective intraperitoneal procedure	Acute Intraperitoneal procedure
Intraperitoneal procedures		
MACE	4 (9%)	0
Hernia repair	13 (29%)	0
Gastrostomy	17 (38%)	0
Other colectomy/colostomy	4 (9%)	0
Urological	7 (16%)	0
Appendicectomy	0	7 (47%)
Cholecystectomy	0	2 (13%)
Exploration/adhesiolysis	0	6 (40%)
Technique for intraperitoneal procedure		
Open	25 (58%)	2 (13%)
Laparoscopic	20 (42%)	13 (87%)
Shunt management at intraperitoneal procedure		
Externalization	1 (2%)	4 (27%)
Wrapped/washed	3 (7%)	1 (7%)
Not specified	42 (93%)	10 (67%)
Days from last shunt procedure to intraperitoneal procedure*	714 ± 803 (Range 13–3403)	819 ± 848 (Range 2–2786)
Intraperitoneal procedure within 30 days of last shunt procedure	4 (9%)	1 (7%)
Total	45	15

^*^Mean ± standard deviation

*MACE* Malone Anterograde Continence Enema

**Table 3:** Elective intra-peritoneal procedure—Cox proportional hazard regression model

Variable	Hazard ratio	95% confidence interval	Significance
Elective (univariate)*	1.18	0.57–2.44	0.66
Elective (multivariate)*	0.91	0.44–1.91	0.80
Age	0.96	0.94–0.99	0.015
Haemorrhagic aetiology	0.93	0.68–1.58	0.66
Orbus sigma valve	1.76	1.24–2.50	0.002

^*^Entered as time-dependent variable

Notably within the emergency cohort (*n* = 15), there was only one case of shunt infection following appendicectomy, although none of these patients underwent prophylactic externalization of their shunt. In the one case of infection, perforation was noted intraoperatively, but the ventriculoperitoneal shunt was not externalized due to the absence of a neurosurgical service at that peripheral hospital, and the infection was identified during a precautionary period of prolonged observation. Additionally, in the cohort undergoing an exploratory procedure without clear preoperative alternative diagnosis or adhesiolysis for small bowel obstruction, 4/6 (66%) of patients subsequently had VP shunt infection identified as the likely primary aetiology of their presentation (three confirmed culture positive, one presumptive). None of these patients had signs or symptoms of meningitis. As for elective procedures, the influence of an emergency intra-peritoneal procedure on shunt survival was assessed as a time-dependent covariate in a Cox-proportional hazard model in both a univariate and multivariate manner. Similarly, no significant effect was found (univariate HR 1.03, 95% CI 0.26–4.19, *p* = 0.96) (Table 4).

**Table 4:** Emergency intra-peritoneal procedure—Cox proportional hazard regression model

Variable	Hazard ratio	95% confidence interval	Significance
Emergency (Univariate)*	1.03	0.26–4.19	0.96
Emergency (Multivariate)*	1.33	0.36–5.42	0.69
Age	0.97	0.94–0.99	0.015
Haemorrhagic aetiology	0.94	0.68–1.29	0.68
Orbus Sigma Valve	1.76	1.24–2.48	0.001

^*^Entered as time-dependent variable

## Discussion

The primary outcome of this study is that the performance of an elective intraperitoneal procedure (whether clean or clean-contaminated) in a patient with a VPS is not associated with a shorter overall survival of that shunt system. Furthermore, in general, no specific precautions were taken to address the presence of a VPS by the general surgical service other than simply noting its presence and making routine efforts to avoid contamination. Secondarily, this study reports a high proportion of shunt infections as the primary aetiology of presentations with an acute surgical abdomen—especially where there is not a clear alternative diagnosis preoperatively. There are relatively few cases of individual pathologies such as appendicitis or cholecystitis in this cohort to make recommendations regarding the management of the VPS system in the setting of those illnesses, as only one case of subsequent shunt infection was identified in a patient who did not have their VPS externalized after a case of perforated appendicitis.

The primary finding is in keeping with some previous literature which found that elective intraperitoneal procedures are safe in the presence of a VPS. This is best studied for gastrostomies (which made up 38% of the intraperitoneal procedures in our cohort) where a meta-analysis of existing literature found that there was no substantial increase in subsequent shunt infection [13]. Importantly, while some authors have reported there may be an increased risk with a close temporal relationship between gastrostomy and shunt placement, none of the gastrostomies in this dataset was inserted less than 13 days after the VPS procedure [13, 14]. Conversely, the one previous attempt to assess the impact of elective intraperitoneal procedures on shunt survival found a marked decrease in shunt survival after elective intraperitoneal procedures were performed after VPS procedures [8]. There were a number of major issues with that analysis including the small sample size, the inclusion of a large cohort of patients with repeated shunt revisions, and the lack of statistical accommodation of the time-dependent nature of the subsequent intraperitoneal procedures. For example, the 20 patients undergoing intraperitoneal procedures had also undergone a total of 118 shunt revisions with a median follow-up of 2 years, which may imply that this sample includes a high-risk cohort of ‘problem shunts’ with a high frequency of revisions. Indeed, their included control group of ‘extraperitoneal’ general surgical operations (consisting predominantly of line insertions) also had a median overall shunt survival of only 6 months, far below the reported medians in most cohorts (e.g. 6.2 years) [15]. The cohort presented herein consists largely of patients after undergoing a first VPS (*n *= 362), and when the analysis is repeated with the exclusion of patients with shunt revisions, the result remains unchanged. These demographic factors, combined with the use of a more appropriate statistical method, comparatively strengthen the findings in this dataset and ensure it is more readily generalized to other practices.

Given only 15 emergency intraperitoneal procedures were performed in our cohort, the absence of a statistically significant relationship between earlier shunt failure and these procedures may reflect that the analysis is simply underpowered and therefore these results are presented descriptively. While little comment can be made to compare or recommend specific management strategies due to the low event rate, there remain a few clinically significant insights. Most critically, 4/15 (27%) of patients with a surgical acute abdomen and a VPS in our series were found to have VPS infection as the primary aetiology, despite no clinical evidence of meningitis at the time. Most concerning was the patients without a clear preoperative diagnosis and undergoing exploratory laparotomy/adhesiolysis for small bowel obstruction, where shunt infection was determined to be the aetiology in 4/6 (66%) of patients. This was consistent with some previous literature which reported that in 50% of patients presenting with an acute abdomen and a VPS in situ, shunt infection was found to be the primary aetiology of the presentation despite initial misdiagnosis in most cases [16]. Although these situations are rare, there is a significant clinical lesson to be considered from these results: In a patient with VPS, an acute abdomen, and no clear alternative diagnosis on abdominal imaging, it is probable that shunt infection is the underlying cause—even in the absence of signs/symptoms of meningitis.

While the prior described relative strengths of this manuscript relative to previous literature include the larger sample size and appropriate statistical methodology, there are several important limitations. Most importantly, it is possible that despite the population-level sample size of this study, it may remain underpowered to detect a significant difference in the primary outcome of shunt survival in patients with and without elective intraperitoneal procedures. The primary barrier to determining whether this study is adequately powered lies in the absence of a readily estimated effect size. If the effect size were calculated based on the effect size from previously discussed data from Li Ching et al. (Hazard ratio 1.55), then 46 patients would be required in the intraperitoneal surgery cohort to be adequately powered to detect a 20% difference with an alpha of 0.05—in which case this cohort would be approximately adequate for the elective surgery but not the emergency surgery cohort [8]. Regardless, these results should be interpreted with caution and may have greatest value when included in a future meta-analysis with other comparable results to allow more detailed subgroup analysis. As in previous efforts, this cohort included cases with relatively minor peritoneal breaches such as gastrostomy and hernia repairs (with opening of the hernia sac). If these cases were excluded, there was still no significant association with earlier shunt failure detected; however, the aforementioned power calculation may not be applicable. A subgroup analysis at the level of individual procedures would require a sample size of thousands of ventriculoperitoneal shunt patients. Such scale is difficult to achieve in a direct analysis of medical records and may be best achieved through an analysis of billing/coding databases or registries.

Additionally, as a retrospective study, this series suffers from the intrinsic issues of retrospective interpretation of medical records, which may be particularly problematic when reliant on documentation of neurosurgical points of interest—such as signs of shunt dysfunction/infection by surgeons of an outside speciality. It is also not possible to be certain what advice/guidance may have been conveyed perioperatively by the neurosurgical service to influence the management of these patients around their intraperitoneal procedures, where such conversations were not documented in the medical record.

Finally, the generalization of these results requires consideration of the context. The study institution is a relatively new developed-world paediatric hospital established 10 years prior to this analysis, which explains the predominance of new ‘virgin’ shunt systems in this cohort. Therefore, these results may not necessarily be similar in the cohort of a more established unit, in an adult practice, or in the developing world, where event rates for shunt failures, abdominal procedures, and infections may be substantially different.

## Conclusion

In a 10-year cohort of paediatric ventriculoperitoneal shunt patients, elective intraperitoneal procedures do not appear to adversely influence shunt survival. In the setting of a patient with a VPS presenting with an acute surgical abdomen, there is a high probability of VPS infection as the primary aetiology.

## Data Availability

No datasets were generated or analysed during the current study.
